# Olfactomedin-4^+^ neutrophils exacerbate intestinal epithelial damage in *Clostridioides difficile* infection

**DOI:** 10.1128/iai.00229-25

**Published:** 2026-01-07

**Authors:** S. Jose, A. Huber, A. Kassam, K. N. Weghorn, M. Powers-Fletcher, D. Sharma, A. Mukherjee, M. N. Alder, R. Madan

**Affiliations:** 1 Division of Infectious Diseases, Department of Internal Medicine, Cincinnati Children’s Hospital Medical Center, Cincinnati, Ohio, USA; 2 Division of Immunobiology, Cincinnati Children’s Hospital Medical Center, Cincinnati, Ohio, USA; 3 Department of Surgery, University of Cincinnati College of Medicine 12303, Cincinnati, Ohio, USA; 4 Division of Pathology and Laboratory Medicine, University of Cincinnati College of Medicine 12303, Cincinnati, Ohio, USA; 5 Department of Pediatrics, University of Cincinnati College of Medicine 12303, Cincinnati, Ohio, USA; 6 Division of Critical Care Medicine, Cincinnati Children’s Hospital Medical Center, Cincinnati, Ohio, USA; 7 Division of Gastroenterology, Hepatology and Nutrition, Cincinnati Children’s Hospital Medical Center, Cincinnati, Ohio, USA; 8 Veterans Affairs Medical Center, Cincinnati, Ohio, USA; University of Illinois Chicago, Chicago, Illinois, USA

**Keywords:** *C. difficile *infection, neutrophils, olfactomedin-4, single-cell transcriptomics, innate immunity

## Abstract

Neutrophils are dominant cells during acute immune response to *Clostridioides difficile* infection (CDI). A higher number of infiltrating colonic neutrophils is clearly linked to greater tissue damage and severe CDI (3, 4). However, the mechanism(s) by which neutrophils exacerbate tissue damage in CDI remain unknown. We investigated the role of a neutrophil subset marked by Olfactomedin-4 expression (OLFM4^+^ neutrophils) during CDI. Single-cell transcriptomics reveal that *Olfm4* is increased in blood neutrophils of infected mice, and these cells exhibit gene signatures characterized by high expression of degranulation genes. In *C. difficile*-infected mice, OLFM4^+^ neutrophils aggregate to areas of severe intestinal epithelial cell (IEC) damage, and plasma OLFM4 was significantly increased in both *C. difficile*-infected mice and patients. *In vitro*, OLFM4^+^ neutrophils and recombinant OLFM4 protein exacerbated *C. difficile* toxin-induced IEC damage. In sum, our studies provide novel insights into neutrophil-mediated pathology and highlight the role of OLFM4^+^ neutrophils in worsening CDI-induced IEC damage.

## INTRODUCTION

*Clostridioides difficile* infection (CDI) is the most common healthcare-associated infection in the US (1). Multiple studies have shown that an increased number of neutrophils in peripheral blood (PB) and colon during acute CDI, along with the persistence of these cells in the colonic lumen over time, is associated with prolonged diarrhea and more severe clinical disease (2–8). Although neutrophil heterogeneity has been studied in other infections and non-infectious inflammatory conditions (9–11), there are no studies that have examined neutrophil heterogeneity in CDI. Furthermore, the mechanisms by which neutrophils worsen CDI are not well understood. After an infectious insult, neutrophils rapidly migrate to the disease site, where their activation triggers degranulation and release of pre-formed proteins from intra-cytoplasmic granules (12). While these neutrophil-derived effectors primarily target pathogens, they can have off-target effects that damage host tissue (11). Since neutrophilia is a hallmark of CDI and is associated with more severe disease (5, 7, 8, 13), we posit that neutrophils and/or neutrophil-derived molecules worsen CDI by contributing to tissue damage.

Olfactomedin 4 (OLFM4) is a member of the olfactomedin-domain containing protein family that was initially identified as a granulocyte colony-stimulating factor-induced gene during myeloid lineage development (14). OLFM4 is expressed in multiple tissues, including prostate, bone, breast, and gastrointestinal (GI) tract, and it is involved in various functions like cellular proliferation, adhesion, apoptosis, and in regulating innate immune responses (15–19). In bone marrow (BM) and blood, neutrophils are the dominant *Olfm4*-expressing cell type: in BM, *Olfm4* is expressed in all granulocyte precursors, whereas in peripheral blood, OLFM4 is present in the specific granules of a subset of neutrophils (17–19). OLFM4 plays an important role in GI diseases (20). In inflammatory bowel disease patients, OLFM4 is secreted extracellularly into areas of active inflammation (21), and in mice, OLFM4 deletion (OLFM4^−/−^) leads to heightened cytokine/chemokine production, inflammatory cell infiltration, and worse outcomes after *Helicobacter pylori* infection and dextran sulfate-induced colitis (22, 23).

To gain a deeper understanding of how OLFM4 impacts CDI, we examined the role of OLFM4^+^ neutrophils and recombinant OLFM4 protein during CDI. We show that neutrophils are the main OLFM4-expressing cells after CDI. Leveraging the power of single-cell transcriptomics, we found that *Olfm4*-expressing neutrophils are characterized by gene signatures associated with neutrophil degranulation, and only one gene is co-regulated with *Olfm4* in these neutrophils. Supporting our transcriptomics data, we found that both mice and humans with CDI had higher levels of circulating OLFM4 compared to controls. *In vitro*, OLFM4^+^ neutrophils from mice and recombinant human OLFM4 (rhOLFM4) exacerbate *C. difficile* toxin-induced intestinal epithelial cell (IEC) injury. Together, our findings offer novel insights into neutrophil-mediated pathology in CDI and highlight a pathogenic role for OLFM4^+^ neutrophils in CDI-induced IEC damage.

## RESULTS

### CDI increases the number of OLFM4^+^ neutrophils in the lamina propria

**Fig 1:**
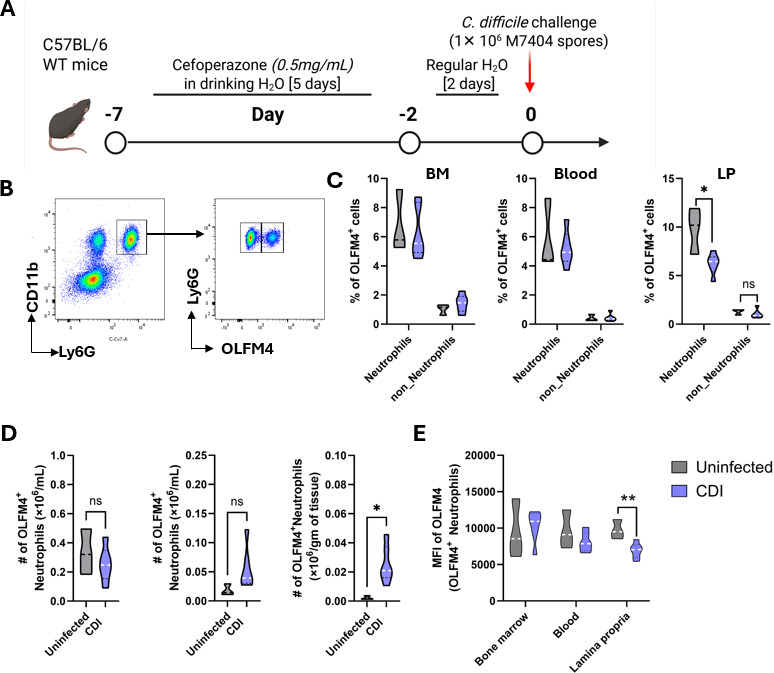
OLFM4^+^ neutrophils are increased in colonic tissue after CDI. (**A**) Schematic representation of the experimental design of CDI in mice. Eight-week-old WT C57BL/6 male mice were pre-treated with antibiotics in drinking water, followed by sham-challenge with phosphate buffered saline (PBS) or infection with 1 × 10^6^ *C. difficile* spores (M7404 strain), and then samples were collected during the acute phase of CDI (1 day after infection). (**B**) Representative flow cytometry plot showing gating strategy to define OLFM4^+^ neutrophils. (**C**) Percentage and (**D**) number of OLFM4^+^ neutrophils in BM, blood, and LP. (**E**) Mean fluorescent intensity of OLFM4 stain in OLFM4^+^ neutrophils in BM, blood, and LP. Data shown as mean ± SEM; *N* = 3 mice in the uninfected group and 6 mice in the infection group and is representative of two independent experiments; *P < 0.05, **P < 0.01; Student’s *t*-test.

While neutrophils are the main *Olfm4-*expressing cell type in the BM and blood of mice and humans (15), in the GI tract, *Olfm4* has been detected in intestinal crypts of the small intestine of both species, and in colonic epithelium of humans (24, 25). To define cells that produce OLFM4 after CDI, we assessed the proportion of OLFM4^+^ cells in the BM, blood, and colon of uninfected and infected wild-type (WT) mice on day 1 after infection by flow cytometry (Fig. 1A and B). In both uninfected and infected mice, OLFM4^+^ cells consistently represented ~8%–10% of neutrophils across all three tissue compartments—BM, blood, and lamina propria (LP)—while OLFM4^+^ cells within the non-neutrophil fraction remained below 2% (Fig. 1C). CDI did not affect the percentage of OLFM4^+^ neutrophils in BM and blood, but their proportions in LP declined (Fig. 1C). While CDI did not impact OLFM4^+^ neutrophil numbers in BM, there was a trend toward increased numbers in the blood and a significant rise in their absolute numbers within the LP following CDI (Fig. 1D). This discordance—an increase in the number but not the proportion of OLFM4^+^ neutrophils in the LP—suggests that their accumulation is not the result of selective recruitment but rather reflects the overall expansion of the neutrophil compartment in the inflamed colon.

OLFM4 is a constituent of specific granules of neutrophils and is released in response to noxious insults (18, 19). Such release may result in both a reduced proportion of OLFM4^+^ neutrophils and a decreased per-cell OLFM4 content, as measured by mean fluorescence intensity (MFI). Consistent with this, we observed a decline in both the percentage of OLFM4^+^ neutrophils and their MFI in the LP following CDI (Fig. 1C through E). Altogether, these data provide compelling evidence that neutrophils are an important source of OLFM4 in mouse BM, blood, and colon after CDI and suggest a scenario whereby CDI induces its extracellular release from neutrophils.

### CDI increases *Olfm4* expression in mature blood neutrophils

**Fig 2:**
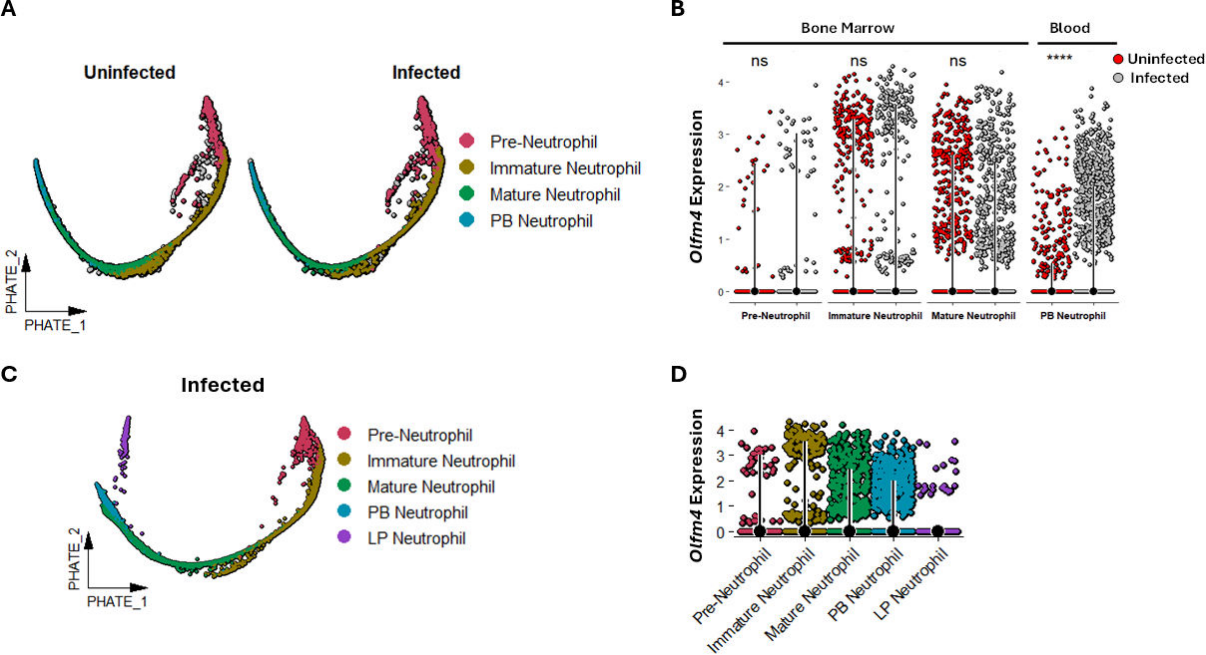
Single-cell transcriptomics of neutrophils reveals increased *Olfm4* gene expression in the PB during acute CDI. (**A**) PHATE plots of BM and blood neutrophils from uninfected and *C. difficile*-infected mice during acute CDI (2 days after infection). Neutrophils are grouped based on maturation state and tissue compartmentalization from pre-neutrophil to immature to mature BM neutrophils and PB neutrophils. (**B**) Geyser plots displaying the comparison of *Olfm4* gene expression of each neutrophil state in different tissues in uninfected and *C. difficile*-infected mice. (**C**) PHATE plots of neutrophils from the infected host only, showing BM, blood, and LP neutrophils. (**D**) Geyser plots displaying *Olfm4* expression in neutrophils from various tissue compartments of the infected host. ****P < 0.0001; Student’s *t*-test.

We have generated a single-cell transcriptomics atlas of BM, blood, and colon neutrophils from WT C57BL/6 mice (both uninfected and during acute CDI; Huber et al*.* (26) under review). Here, we leveraged the power of this single-cell RNA sequencing (scRNAseq) data set to perform an in-depth analysis of *Olfm4*-expressing and non-expressing neutrophils. Potential of heat diffusion for affinity-based trajectory embedding (PHATE) analysis was used to visualize BM and blood neutrophil populations across different developmental stages (Fig. 2A). *Olfm4* was expressed in pre-neutrophils, immature neutrophils, and mature neutrophils in BM as well as in mature neutrophils in the blood, with the highest expression detected in immature and mature BM neutrophils (Fig. 2B). CDI did not alter *Olfm4* transcript levels in BM neutrophils; however, in circulating neutrophils, *Olfm4* expression was increased in cells from infected mice (Fig. 2B). A similar increase in *OLFM4* transcripts in circulating leukocytes has been reported in bulk RNA-seq studies comparing *C. difficile*-infected patients with healthy controls (27). Despite this transcriptional upregulation, neither the proportion of OLFM4^+^ neutrophils in blood nor their MFI changed after CDI, suggesting the involvement of post-transcriptional mechanisms regulating OLFM4 protein expression.

At homeostasis, there are very few neutrophils in the LP. Thus, we were unable to get enough cells to perform single-cell transcriptomics in colonic neutrophils from uninfected mice. However, PHATE analysis of BM, blood, and colon neutrophil populations from infected mice revealed that: (i) LP neutrophils were at the end of the developmental trajectory (Fig. 2C); and (ii) while intensity of *Olfm4* expression in LP neutrophils was similar to mature BM and blood neutrophils, compared to mature BM and peripheral blood neutrophils, total number of neutrophils expressing *Olfm4* was fewer in LP (Fig. 2D).

### *Olfm4***-**expressing neutrophils are primed for degranulation and exhibit gene signatures consistent with immature neutrophils

**Fig 3:**
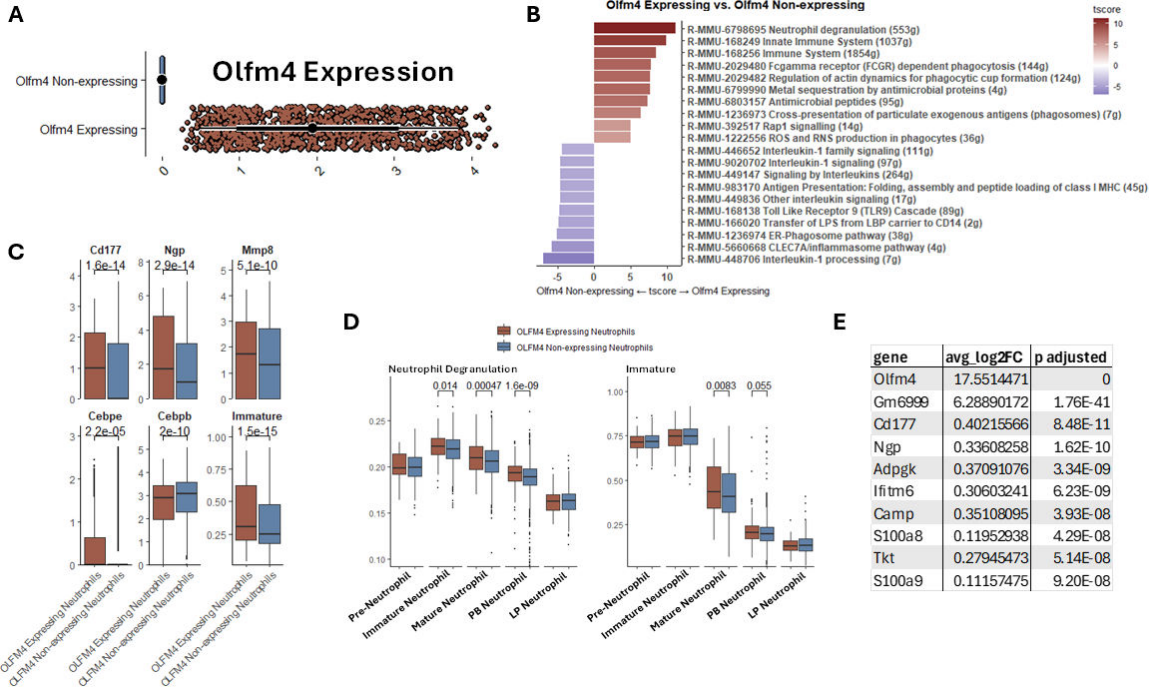
*Olfm4*-expressing neutrophils exhibit gene signatures associated with degranulation and immaturity during acute CDI. (**A**) Geyser plot of *Olfm4* expression after neutrophils was divided into two groups based on *Olfm4* expression: *Olfm4*-expressing and *Olfm4* non-expressing. (**B**) Waterfall plot of GSEA of *Olfm4-*expressing and non-expressing neutrophils using the Reactome database. (**C**) Box plots of genes and immature signature score; student’s *t*-test. (**D**) Neutrophil degranulation and immature neutrophil score of *Olfm4*-expressing and *Olfm4* non-expressing neutrophils grouped by maturation state/tissue compartment. (**E**) Table showing differentially expressed genes (comparison of Olfm4-expressing vs non-expressing cells); Wilcoxon rank sum test with Bonferroni correction.

To characterize transcriptional differences between neutrophils expressing or not expressing *Olfm4*, we performed gene set enrichment analysis (GSEA) of scRNAseq data from BM, blood, and LP neutrophils (of infected host; Fig. 3A and B). Compared to *Olfm4* non-expressing neutrophils, *Olfm4*-expressing neutrophils showed an ~8- to 10-fold upregulation of genes associated with degranulation and the innate immune system, whereas genes related to the *Clec7* inflammasome pathway and IL-1 processing were increased by approximately sevenfold in *Olfm4* non-expressing cells (Fig. 3B). Upon further investigation, we found that enrichment of the neutrophil degranulation pathway in *Olfm4*-expressing neutrophils was driven by elevated expression of *Cd177*, *Ngp*, and *Mmp8* (Fig. 3C). Although peak expression of neutrophil degranulation genes occurs in immature neutrophils, comparison between *Olfm4*-expressing and non-expressing neutrophils showed that the degranulation gene signature was significantly upregulated in immature and mature BM and PB neutrophils of the *Olfm4*-expressing group (Fig. 3D).

CCAAT enhancer-binding protein epsilon and beta (CEBPε and CEBPβ) are transcription factors that play a key role in various stages of neutrophil development: CEBPε is involved in early stages of granulopoiesis, while CEBPβ is a regulator of late myeloid differentiation (9). Together, BM, blood, and LP *Olfm4*-expressing neutrophils expressed significantly higher *Cebpe*, but few *Cebpb* transcripts compared to *Olfm4* non-expressing cells (Fig. 3C). These data suggested that *Olfm4-*expressing neutrophils might be less mature. Thus, we used a gene signature score that characterizes immature neutrophils (defined in Xie et al. [9]) to compare scRNAseq data from these populations. Indeed, *Olfm4*-expressing neutrophils had a significantly higher immature neutrophil gene signature score, compared to *Olfm4* non-expressing neutrophils (Fig. 3C). However, when gene expression was stratified by maturation state/tissue compartment, it became apparent that this upregulation was predominantly confined to mature *Olfm4*-expressing neutrophils of the BM compared to *Olfm4* non-expressing neutrophils (Fig. 3D). The key genes that contribute to the higher immature gene signature score in mature *Olfm4*-expressing neutrophils included those that have been previously reported to be expressed in pre-neutrophil and immature neutrophil stages (i.e., *Anxa3*, *P*.adj = 0.0075; *Dstn*, *P*.adj = 0.0064; *Cd177*, *P*.adj = 0.0036; and *Anxa1*, *P*.adj = 0.021; green boxes in Fig. S1) (9, 28).

Differential expression testing uncovered another very interesting feature. *Olfm4*-expressing cells had higher transcript levels for several genes encoding granule and cytoplasmic proteins (i.e., *Cd177*, *Ngp*, *S100a8*, and *S100a9*) (29, 30). They also showed increased expression of genes involved in antimicrobial peptide synthesis (i.e., *Camp*), regulation of cell-cell adhesion and differentiation (i.e., *Ifitm6*), and metabolic pathways (i.e., *Adpgk* and *Tkt*) (31, 32). However, the largest difference when compared to *Olfm4*-non-expressing neutrophils was only in two genes, both of which were >5 log fold increased in *Olfm4*-expressing and were unique to this subset (Fig. 3E). These genes were *Olfm4*, which would be expected, and a gene in the house mouse (*Mus musculus*), called *Gm6999* (33). Notably, *Gm6999* encodes for a long non-coding RNA (lncRNA), and its genomic location is on the same chromosome as *Olfm4* at a site that overlaps with the 5′ region of the mouse *Olfm4* locus (33). Although *Gm6999* function has not yet been fully characterized, lncRNAs play an important role in chromatin remodeling and epigenetic regulation (34), warranting further investigation. Together, these data reveal transcriptional differences between *Olfm4*-expressing and non-expressing neutrophils, including signatures associated with degranulation and immaturity.

### OLFM4^+^ neutrophils exhibit differential CXCR4 expression

**Fig 4:**
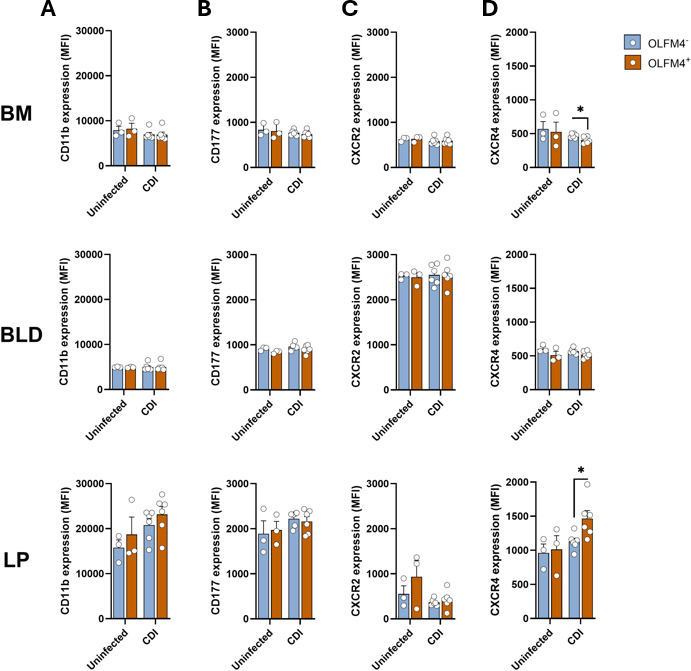
CXCR4 expression changes in OLFM4^+^ neutrophils after CDI. (**A–D**) MFI of (**A**) CD11b, (**B**) CD177, (**C**) CXCR2, and (**D**) CXCR4 in OLFM4^+^ and OLFM4^−^ neutrophils in BM, blood, and LP of uninfected and *C. difficile*-infected mice during acute CDI. Data shown as mean ± SEM; *N* = 3 mice in the uninfected group and 6 mice in the infection group. *P < 0.05; Student’s *t*-test.

In addition to transcriptomics, neutrophil surface proteins have been used to characterize different sub-populations (30, 35, 36). Therefore, to further characterize OLFM4^+^ and OLFM4^−^ neutrophils, we stained these cells with fluorescently conjugated antibodies to CD11b, CD177, CXCR2, and CXCR4 and performed flow cytometry (Fig. 4). These markers were selected because they provide functional insights into neutrophil activation, trafficking, and localization. Specifically, CD11b and CD177 are associated with neutrophil activation and degranulation, while CXCR2 and CXCR4 regulate neutrophil migration between the BM, circulation, and inflamed tissues (36). Assessing the expression of these markers enables us to determine whether OLFM4 expression identifies neutrophil subsets with distinct activation states or migratory potential during *C. difficile* infection. In uninfected mice, there was no difference in MFI of CD11b, CD177, CXCR2, or CXCR4 between OLFM4^+^ and OLFM4^−^ neutrophils (Fig. 4). During acute CDI, while CD11b, CD177, and CXCR2 MFI were similar on both OLFM4^+^ and OLFM4^−^ neutrophils in all three tissues, CXCR4 MFI was different (Fig. 4A through C). In BM, OLFM4^+^ neutrophils had lower CXCR4, but in LP, these cells had higher CXCR4, compared to OLFM4^−^ neutrophils in the respective tissues (Fig. 4D). Published studies show that reduced CXCR4 drives mobilization of BM neutrophils into bloodstream (36), whereas at tissue sites, CXCR4 is a marker of aged neutrophils that can contribute to tissue damage (37). Thus, our data point to a scenario where acute CDI increases mobilization of OLFM4^+^ neutrophils from the BM, and in LP, these cells exhibit a hyper-inflammatory phenotype.

### OLFM4^+^ neutrophils exacerbate *C. difficile* toxin-induced IEC injury

**Fig 5:**
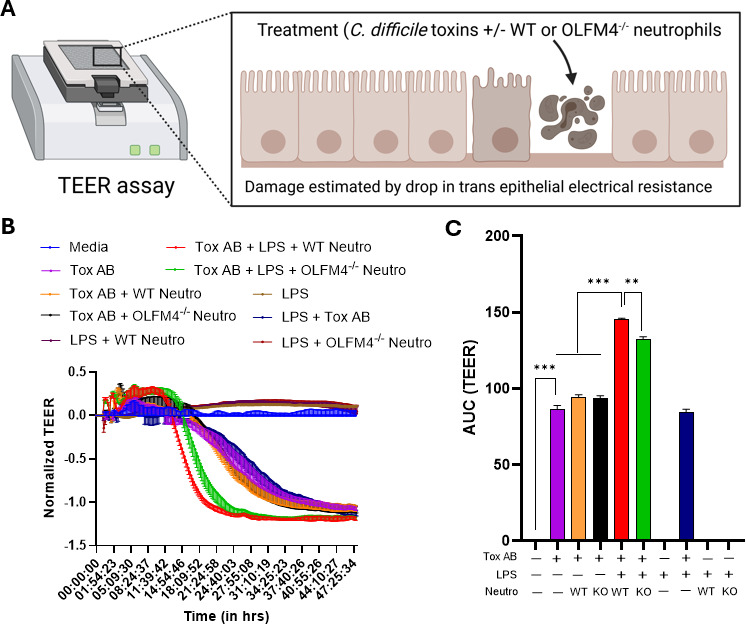
OLFM4^+^ neutrophils exacerbate *C. difficile* toxin-induced epithelial damage. (**A**) Schematic representation of the TEER assay. (**B**) Change in TEER of mouse epithelial cell (CMT-93) monolayers incubated with *C. difficile* toxins A and B, LPS, and neutrophils over 48 h. (**C**) Area under the curve of the TEER plots. Curves are plotted as the normalized mean value of three replicates, representative of two independent experiments; bar graph data shown as mean ± SEM. **P < 0.01 and ***P < 0.001; one-way repeated measures analysis of variance (ANOVA) with Tukey’s multiple comparisons test. The schematic in (**A**) was created in BioRender. Jose, S. (2025) https://BioRender.com/njcsoxt.

A high proportion of OLFM4^+^ neutrophils in the blood correlates with severe end-organ damage and worse outcomes (38–40). Thus, we tested the direct effect of neutrophil OLFM4 expression on *C. difficile*-associated epithelial injury. BM neutrophils from WT and age-matched OLFM4^−/−^ mice were FACS sorted to obtain highly pure populations (>95% purity). Neutrophils were then cultured with a mouse epithelial cell line (CMT-93 cells) with or without *C. difficile* toxins and lipopolysaccharide (LPS) in a transepithelial electrical resistance (TEER) assay plate (Fig. 5A). As expected, *C. difficile* toxins caused damage to the IEC barrier as seen by a reduction in TEER (Fig. 5B and C). LPS alone or the addition of LPS with neutrophils did not adversely affect the IEC barrier. However, in combinations of *C. difficile* toxins with LPS and neutrophils, toxin-induced drop in TEER was further exacerbated in the presence of WT neutrophils (26), but not when OLFM4^−/−^ neutrophils were used (Fig. 5B and C). Altogether, our results are congruent with published studies that show a correlation of OLFM4^+^ neutrophil numbers with end-organ damage and support a disease-enhancing role for OLFM4^+^ neutrophils (at least *in vitro*) via adverse impact on IEC barrier integrity.

### OLFM4 preferentially aggregates at sites of *C. difficile* toxin-induced IEC injury

Our flow cytometry data showed that the percentage of OLFM4^+^ neutrophils was similar in naïve and *C. difficile*-infected mice across all compartments except colonic tissue, where the proportion was reduced in *C. difficile-*infected mice compared to uninfected controls (Fig. 1C). Activated neutrophils are known to release OLFM4 as part of neutrophil extracellular traps (NETs) (18). Our transcriptomic analysis showed that *Olfm4*-expressing neutrophils upregulated genes associated with degranulation. We therefore hypothesized that OLFM4 is released from neutrophils at sites of inflammation or tissue injury, decreasing intracellular OLFM4 and thus lowering the proportion of OLFM4^+^ neutrophils detected in tissue.

**Fig 6:**
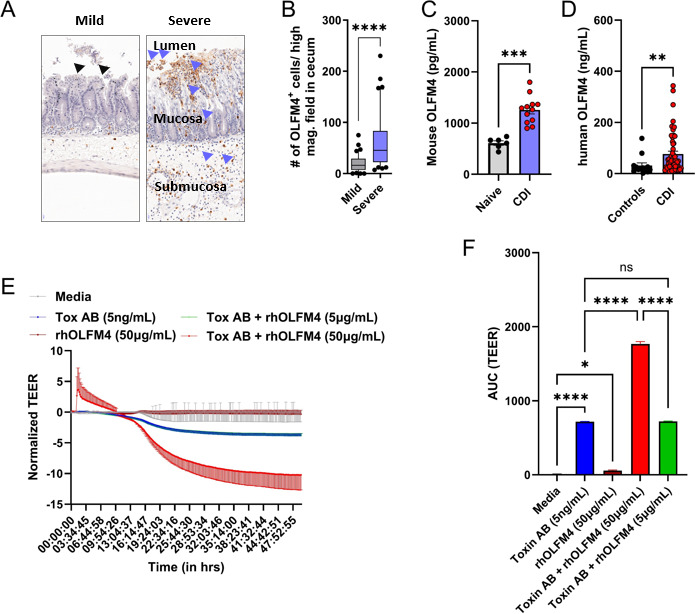
OLFM4 aggregates to areas of epithelial damage during acute CDI, and rhOLFM4 exacerbates *C. difficile* toxin-induced IEC damage. (**A**) Representative images of immunohistochemistry staining for OLFM4 in areas of mild vs severe IEC in cecal sections of *C. difficile*-infected mice on day 1 after infection. (**B**) Number of OLFM4^+^ cells per high magnification field in cecal sections; *N* = 6 mice per group; 30–40 fields counted. (**C**) OLFM4 levels in mouse serum collected during the acute phase of CDI; *N* = 6–12 mice per group. (**D**) OLFM4 levels in human plasma collected from CDI patients and controls (patients with diarrhea without CDI whose WBC count is within normal range); *N* = 13 controls and 53 CDI patients. (**E**) Change in TEER of Caco2 cells incubated with *C. difficile* toxins and rhOLFM4 over 48 h. (**F**) Area under the curve of TEER plots. Curves are plotted as the normalized mean value of three replicates, representative of two independent experiments. Bar graph data shown as mean ± SEM; *P < 0.05, **P < 0.01, ***P < 0.001, and ****P < 0.0001; tudent’s *t*-test.

Immunohistochemistry of colonic tissue revealed OLFM4^+^ cells distributed throughout the submucosa during acute CDI, with more intense staining in regions of severe colonic damage (Fig. 6A; Fig. S3; blue arrowheads), compared to areas with milder injury (Fig. 6A; Fig. S3; black arrowheads). Mice with severe epithelial damage scores (score >2.5 on a scale of 0–4) had more OLFM4^+^ cells per high-power field than those with less injury (Fig. 6B). Furthermore, indicating OLFM4 protein is possibly released from activated colonic neutrophils, *C. difficile-*infected mice exhibited higher serum OLFM4 levels compared to uninfected controls during the acute phase of disease (Fig. 6C), and these levels correlated with circulating neutrophil counts (Fig. S2A). Notably, compared to sham-challenged mice, the MFI of intracellular OLFM4 was reduced in colonic neutrophils of *C. difficile*-infected mice (Fig. 1E). Together, these findings suggest that OLFM4^+^ neutrophils are primed for degranulation during CDI, leading to extracellular release of OLFM4 at sites of inflammation.

### Recombinant human OLFM4 protein aggravates *C. difficile*-toxin-induced IEC damage

We next quantified serum OLFM4 concentration in patients with acute CDI and compared them to individuals with diarrhea who tested negative for *C. difficile*. Similar to our murine model, CDI patients had significantly higher systemic OLFM4 levels than controls (Fig. 6D), and these levels correlated with circulating WBC counts (Fig. S2B). Previous studies have shown that both the proportion of OLFM4^+^ neutrophils and the amount of circulating OLFM4 protein are associated with worse outcomes in a variety of infections, including viral (dengue, influenza, and RSV), bacterial (*H. pylori*, *Escherichia coli*, and *Staphylococcus aureus*), and parasitic (malaria) infections (15, 38, 39, 41, 42). In sepsis, a high percentage of OLFM4^+^ neutrophils are found in those who have severe end-organ damage (40). While OLFM4^+^ and OLFM4^−^ neutrophils exhibit similar activation and phagocytic capacities, they differ in their propensity for NET formation (19). It was reported that OLFM4 release from neutrophils can be induced by stimulation of the degranulation pathway and also via NETosis (19). While OLFM4 release after degranulation needed a strong secretagogue, OLFM4 was more readily released as part of NET components (43).

To determine the role of OLFM4 protein in CDI pathogenesis, we used recombinant human OLFM4 (rhOLFM4) on IECs: human epithelial cells (Caco2 cells) were incubated with rhOLFM4 in the presence and absence of *C. difficile* toxins, and TEER was measured, as described above. As expected, *C. difficile* toxins caused IEC barrier damage, and TEER was significantly reduced compared to the media alone control (Fig. 6E and F). rhOLFM4 protein alone (even at a high concentration of 50 µg/mL) did not impact IEC barrier integrity. However, when co-administered with toxins at the same concentration, rhOLFM4 significantly exacerbated barrier disruption (Fig. 6E and F). This additive effect of rhOLFM4 was not observed at a lower concentration (5 µg/mL rhOLFM4; Fig. 6E and F).

## DISCUSSION

The most important findings from our studies are that: (i) CDI augments *Olfm4* in peripheral blood neutrophils; (ii) *Olfm4*-expressing neutrophils are enriched for degranulation-associated gene signatures; and (iii) OLFM4^+^ neutrophils, as well as recombinant OLFM4 protein, worsen *C. difficile* toxin-induced IEC damage. In sum, these data suggest that OLFM4 is a marker of pathogenic neutrophil subsets and imply a role for extracellularly released OLFM4 in worsening CDI via effect on the IEC barrier. In cell cultures, neutrophils isolated from WT mice demonstrated a greater capacity to exacerbate IEC damage caused by pathogen-derived factors (i.e., *C. difficile* toxins), compared to neutrophils from OLFM4^−/−^ mice. Of note, OLFM4 marks a subset of neutrophils, and in C57BL/6 mice, only ~7%–10% neutrophils express OLFM4 (18). Thus, the difference seen in TEER drop between WT and OLFM4^−/−^ neutrophils (Fig. 5) suggests that even small proportions of these cells may be able to affect IEC damage. Previous studies have raised the possibility of using circulating OLFM4^+^ neutrophil percentage and/or its serum level as a biomarker to predict clinical disease severity after infections (38, 40, 42). Our observations that CDI patients had more serum OLFM4 compared to uninfected individuals, and that rhOLFM4 directly aggravated IEC damage caused by *C. difficile* toxins, further highlight the importance of this molecule in clinical CDI. While the additive effect of rhOLFM4 on *C. difficile* toxin-induced IEC damage was seen only at high concentrations, locally high concentrations of neutrophil granule proteins have been reported at sites of tissue damage (44–46). The clinical spectrum of CDI ranges from mild, self-limited diarrheal illness to severe colitis and sometimes death. Although there are likely many drivers of inter-individual variability in CDI severity, one contributor could be the proportion of OLFM4^+^ neutrophils and/or serum OLFM4 concentration. It is well known that the proportion of OLFM4^+^ neutrophils is 5%–50% in humans (17, 43), and this protein is released extracellularly (19). Thus, differences in OLFM4^+^ neutrophil percent prior to CDI could impact the amount that is released extracellularly in response to infection, with higher amounts resulting in worse disease. In fact, extracellular OLFM4 concentration (serum OLFM4) in our murine model and patient data set was associated with neutrophil and WBC count, respectively. Together, the data presented here support an injury-associated function of OLFM4^+^ neutrophils and rhOLFM4, laying the groundwork for a future study to prospectively examine the impact of the percentage of OLFM4^+^ neutrophils and the serum concentration of OLFM4 on CDI severity.

scRNAseq provides high-resolution data that identifies complex biological pathways and allows for a deeper understanding of cellular diversity among populations (9, 10, 35). We observed increased *Olfm4* transcripts in peripheral blood neutrophils from infected hosts compared to uninfected controls. Notably, immature and mature BM, as well as circulating *Olfm4*-expressing neutrophils, upregulated genes enriched in immature neutrophil states, in contrast to their *Olfm4*-negative counterparts. These *Olfm4*-positive neutrophils also displayed enhanced expression of genes associated with degranulation, a program most strongly expressed in immature neutrophils. These findings are consistent with prior studies demonstrating that inflammation can shorten the window of post-mitotic maturation in the BM, resulting in the release of transcriptomically less mature neutrophils into circulation (9, 28, 47). In our data set, these observations are specific to the infected host, and thus, they define CDI-driven transcriptional signatures in neutrophils and expand our understanding of neutrophil heterogeneity after a bacterial infection.

Although a large number of BM neutrophils express *Olfm4* during myelopoiesis, OLFM4 protein is restricted to only a subset of mature blood neutrophils (17, 43). This observation suggests that post-transcriptional modification is a key regulator of the OLFM4 protein. However, the underlying mechanisms remain unknown. Although it was detected in a minor fraction of *Olfm4*-expressing neutrophils, *Gm6999* was not expressed at all in other neutrophils. *Gm6999* encodes for an lncRNA, and its locus on chromosome 14 overlaps *Olfm4*. The function of *Gm6999* has not been defined yet, but lncRNAs are critical in transcriptional and post-transcriptional gene regulation (34). Thus, our data raise the possibility that lncRNA *Gm6999* could be a regulator of *Olfm4* during neutrophil development and differentiation.

Despite making key observations on neutrophil programming, heterogeneity, and its role in driving CDI-associated IEC injury, our studies do have some limitations. The mechanisms by which OLFM4 neutrophils and/or OLFM4 protein exacerbate CDI-associated damage remain unknown and would be future areas of interrogation. OLFM4 interacts with frizzled receptors, a vital component of the Wnt/β-catenin pathway involved in proliferation and survival of IECs (48). Thus, OLFM4 could directly affect the repair of the IEC barrier in CDI; however, whether OLFM4 augments pathways of cell damage, inhibits IEC repair and regeneration, or affects both remains unknown and will be a focus of future studies. OLFM4 is a constituent of specific granules that is released through both conventional (i.e., degranulation) and non-conventional (i.e., part of NETs) secretion pathways (17, 18). Our transcriptomic analyses indicate upregulation of degranulation-associated genes in *Olfm4*-expressing neutrophils compared to non-expressing neutrophils, suggesting that OLFM4 release during CDI may occur via degranulation, leading to a reduced proportion of OLFM4^+^ neutrophils in tissue. This is supported by increased extracellular OLFM4 staining in areas of mucosal damage (Fig. 6A; Fig. S3), which coincides with the decline in OLFM4^+^ neutrophil proportion (Fig. 1E), as well as elevated plasma OLFM4 levels in CDI-infected mice. The precise mechanism of OLFM4 release will be addressed in future studies by assessing intracellular and extracellular OLFM4 levels following *ex vivo* stimulation of LP neutrophils in the presence or absence of degranulation inhibitors.

Severe CDI is a major clinical problem that responds poorly to current therapies. Finding new markers of severe CDI will greatly assist in identifying patients who are at a higher risk of developing severe disease. This study provides crucial experimental evidence of a pathogenic role for OLFM4^+^ neutrophil population in CDI and is an important first step in understanding the relationship between OLFM4 and CDI severity, which can provide valuable insights into the pathogenesis and prognosis of this disease.

## MATERIALS AND METHODS

### Mouse model of CDI and histology

Animal studies were conducted at the Veterinary Medicine Unit at VAMC Cincinnati, under pathogen-free conditions. Eight- to thirteen-week-old WT C57BL/6 mice were challenged with *C. difficile* spores (M7404 1 × 10^6^/mouse; or VPI 10463 1 × 10^4^/mouse, only for Fig. 6A and B) by oral gavage, as described previously (49, 50). To prevent cross-infection, mice were housed individually after CDI. For histopathological analysis, cecal tissues were fixed in Bouin’s solution, embedded in paraffin, sectioned, and stained with hematoxylin and eosin. Sections were scored to evaluate inflammation, edema, and epithelial disruption (range of 0–4 for each parameter) by a pathologist in a blinded fashion, as previously described (49, 50).

For OLFM4 staining, formalin-fixed, paraffin-embedded tissue sections were deparaffinized, rehydrated, and subjected to antigen retrieval in citrate buffer. Endogenous biotin was blocked with an Avidin/Biotin Blocking Kit (Biocare Medical), and peroxidase activity was quenched with Bloxall (Vector Laboratories). Sections were incubated with rabbit anti-mouse OLFM4 primary antibody, followed by secondary antibody staining. Slides were digitally scanned, and OLFM4-positive cells were quantified manually at 40× magnification using CaseViewer software.

### Neutrophil isolation and *in vitro* assays

BM neutrophils were sorted from WT and OLFM4^−/−^ mice using a magnetic-activated cell sorting (MACS) mouse neutrophil isolation kit (Miltenyi Biotech #130-097-658, Germany) (8). Their purity and OLFM4 expression were assessed using flow cytometry. Sorted neutrophils were added to CMT-93 cells on a 96-well E-plate (Agilent # 300601010); TEER changes were measured every 15 minutes for 48 h.

### Human sample collection and ELISA

Discarded blood samples were collected from hospitalized patients at the University of Cincinnati (UC) Medical Center: 53 *C*. *difficile* cases (patients with diarrhea and stool TcdB PCR positive) and 13 controls (patients with diarrhea and stool TcdB PCR negative). Samples were collected within 48 h of *C. difficile* testing. Studies were approved by the UC Institutional Review Board; #2019-0195. For mice, blood samples were collected 1 day after infection. Plasma was separated from blood by centrifugation at 5,000 × *g* at 4°C for 5 minutes and stored at −80°C until enzyme-linked immunosorbent assay (ELISA) use. Plasma OLFM4 levels were measured using human (abcam; Cat# ab267805) and mouse (LSBio; Cat# LS-F6064-1) ELISA Kits as per manufacturer’s recommendation.

### scRNAseq

BM, blood, and colon from age-matched uninfected and *C. difficile*-challenged mice were collected on day 2 after infection. Neutrophils were sorted using MACS negative selection beads (BD Biosciences) as previously described (8, 50). RNase inhibitor (1 U/µL) was added to the neutrophil sorting buffer to inhibit RNase activity; for colon samples, mouse CD326 (EpCAM) antibodies were added to the neutrophil sorting kit to reduce the amount of IEC contamination. Cells were then resuspended in media with 1 U/µL RNase inhibitor and 10% fetal calf serum (FCS) at room temperature. Cell viability was determined by trypan blue exclusion. All samples sent for sequencing had viability >70%. Sequencing was performed using Chromium X scRNAseq and Illumina platform equipment at the CCHMC Single-Cell Genomics Core. For cell preparation, NextGEM assay was used and sequencing with 3'v3.1 assay chemistry. See supplementary materials for details of scRNAseq data analyses, including post-sequencing filtering, quality control, annotation, clustering, GSEA, etc.

### Statistical analysis

Statistical analyses were performed using GraphPad Prism 5.0 software (GraphPad Software Corporation, Inc, CA, USA) or using R. For comparison of groups, a student’s *t*-test or multiple *t*-tests were used. A *P*-value below 0.05 was considered significant. For comparisons between neutrophil clusters from uninfected and infected mice, statistical differences were calculated with an unpaired student’s *t*-test (*P < 0.05; **P < 0.01; ***P < 0.001; ****P < 0.0001). For differential gene expression (DGE) analysis, differential expression testing was based on the Wilcoxon rank sum test, and Bonferroni correction was used for adjusted *P*-values (Fig. 3D).

## Data Availability

Sequencing data generated for this study are available at https://singlecell.broadinstitute.org/single_cell/study/SCP3376/olfactomedin-4-neutrophils-exacerbate-intestinal-epithelial-damage-in-clostridioides-difficile-infection#study-download.

## SUPPLEMENTAL MATERIAL

Supplemental material iai.00229-25-s0001.docx Fig. S1, S2 and S3; Supplement methods.

ASM does not own the copyrights to Supplemental Material that may be linked to, or accessed through, an article. The authors have granted ASM a non-exclusive, world-wide license to publish the Supplemental Material files. Please contact the corresponding author directly for reuse.
