# Supplementary material for: Olfactomedin-4+ neutrophils exacerbate intestinal epithelial damage in Clostridioides difficile infection
Source: Infect Immun. 2026 Jan 7;94(2):e00229-25. doi: 10.1128/iai.00229-25 (PMC12890028; doi:10.1128/iai.00229-25)
Supplement: Supplemental material — Fig. S1, S2 and S3; Supplement methods. [file iai.00229-25-s0001.docx]

**Supplementary Materials**

**Supplementary Figure 1: Immature neutrophil gene signature of *Olfm4*-expressing and non-expressing neutrophils.** Genes used for immature neutrophil score were plotted in a heatmap of different neutrophil clusters split by *Olfm4* expression. Genes highlighted in green boxes were significantly different between *Olfm4*-expressing and non-expressing neutrophils (Wilcoxon test with multiple testing correction; significance determined using adjusted p-values).


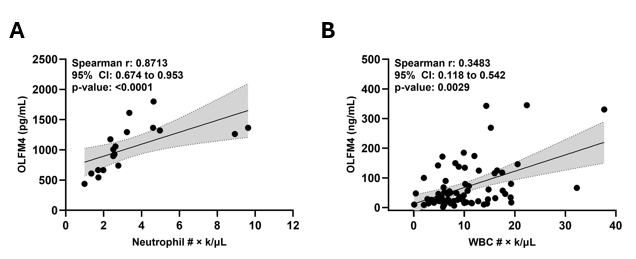


**Supplementary Figure 2:** **Serum OLFM4 levels correlated with blood neutrophils**. Spearman correlation analysis of (A) blood neutrophil counts and serum OLFM4 in *C. difficile*-infected mice in the acute phase of infection, and (B) WBC count and serum OLFM4 levels in *C. difficile* patients.


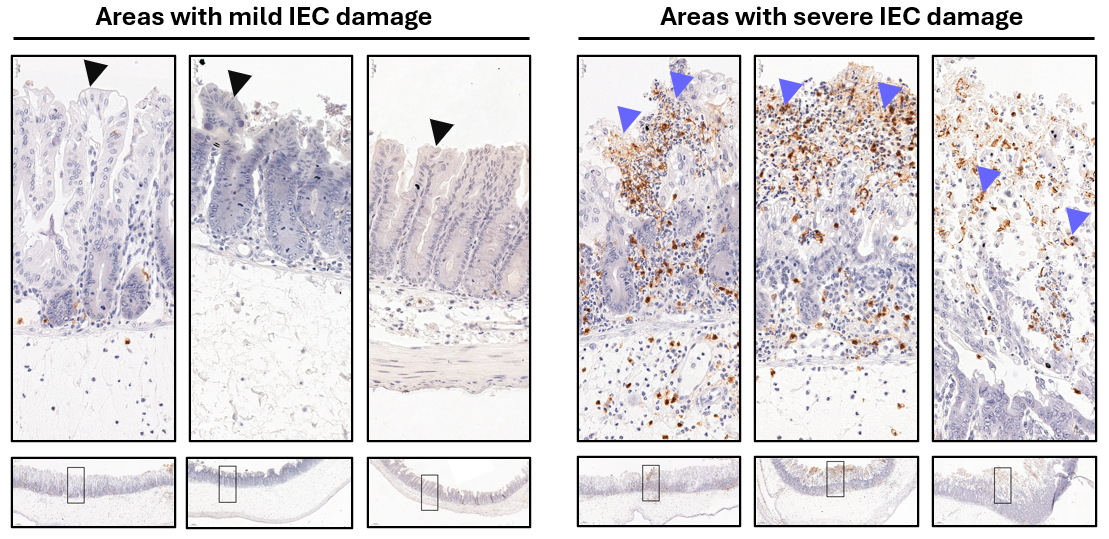
 **Supplementary Figure 3: OLFM4+ neutrophils aggregate to areas of severe intestinal epithelial cell (IEC) damage.** Representative images of immunohistochemistry staining for OLFM4 in areas of mild vs. severe IEC in cecal sections of C. difficile-infected mice on day 1 after infection.

**Supplementary methods**

**Post-Sequencing Filtering and Quality Control**

Cell Ranger 7.0.0 was utilized for sequencing read alignment to the mm10 mouse transcriptome and quantification of the expression of cellular transcripts. Output files were analyzed using Seurat 5.0^1^ in which filtering data set for low quality cells, doublets, cells with mitochondrial, hemoglobin, and ribosomal genes, and non-neutrophils was performed.

**Annotation and Clustering**

Annotation of neutrophils was performed by using: (i) AUCell and neutrophil-specific gene lists from PangloaDB; (ii) SingleR to validate clusters with Tabula Muris Senis and ImmGen dataset; and (iii) ToppGene database^2-5^. Neutrophils of BM and blood of uninfected and infected hosts were integrated and clustered using Seurat. Based on gene expression of TFs (*Cebpe* and *Cebpb*), granule protein genes, chemotaxis genes, and cell cycle/proliferative states, we annotated the neutrophil clusters as pre-neutrophil, immature neutrophil, mature neutrophil, and mature PB neutrophils as defined in Xie et. al. 2020^6^. For analysis of the infected host, neutrophils from the LP of the colon were termed “LP Neutrophil.” Phate analysis of neutrophil subsets was performed using PhateR^7^.

**Single-Cell Data Visualization**

All plots generated for single-cell RNAseq analyses were generated using ScPubR and SeuratExtend^8^.

**Differential Gene Expression Analysis**

After subsetting neutrophils based on presence or absence of *Olfm4* transcripts (using an expression level of 0.01 as a cutoff), we found gene signatures of *Olfm4-*expressing/non-expressing neutrophils using FindAllMarkers.

**Gene Set Enrichment Analysis**

Gene set enrichment analysis was performed using SeuratExtend to define pathways upregulated in *Olfm4-*expressing and non-expressing neutrophils using the Reactome database^9^. Gene set enrichment for immature neutrophil–associated genes was calculated using the AUCell package and plotted in Seurat Extend^3^. Immature neutrophil gene list (G3) was obtained from Xie et. al. 2020 supplemental files^6^.

**Reference:**

1 Hao, Y. *et al.* Dictionary learning for integrative, multimodal and scalable single-cell analysis. *Nat Biotechnol* **42**, 293-304 (2024). <https://doi.org/10.1038/s41587-023-01767-y>

2 Franzen, O., Gan, L. M. & Bjorkegren, J. L. M. PanglaoDB: a web server for exploration of mouse and human single-cell RNA sequencing data. *Database (Oxford)* **2019** (2019). <https://doi.org/10.1093/database/baz046>

3 Aibar, S. *et al.* SCENIC: single-cell regulatory network inference and clustering. *Nat Methods* **14**, 1083-1086 (2017). <https://doi.org/10.1038/nmeth.4463>

4 Aran, D. *et al.* Reference-based analysis of lung single-cell sequencing reveals a transitional profibrotic macrophage. *Nat Immunol* **20**, 163-172 (2019). <https://doi.org/10.1038/s41590-018-0276-y>

5 Tabula Muris, C. A single-cell transcriptomic atlas characterizes ageing tissues in the mouse. *Nature* **583**, 590-595 (2020). <https://doi.org/10.1038/s41586-020-2496-1>

6 Xie, X. *et al.* Single-cell transcriptome profiling reveals neutrophil heterogeneity in homeostasis and infection. *Nat Immunol* **21**, 1119-1133 (2020). <https://doi.org/10.1038/s41590-020-0736-z>

7 Moon, K. R. *et al.* Visualizing structure and transitions in high-dimensional biological data. *Nat Biotechnol* **37**, 1482-1492 (2019). <https://doi.org/10.1038/s41587-019-0336-3>

8 Cao, J. *et al.* The single-cell transcriptional landscape of mammalian organogenesis. *Nature* **566**, 496-502 (2019). <https://doi.org/10.1038/s41586-019-0969-x>

9 Hua, Y. L. W., Zhao F., Rambow, F.

<https://www.biorxiv.org/content/10.1101/2024.08.01.606144v1>
